# Malignant Risk Assessment of Cystic-solid Thyroid Nodules Based on Multimodal Ultrasound Features: A Systematic Review and Meta-analysis

**DOI:** 10.2174/0115734056360924250106091630

**Published:** 2025-01-14

**Authors:** Rongwei Liu, Hua Chen, Jianming Song, Jun Ye

**Affiliations:** 1 Department of Medical Ultrasound, The First Affiliated Hospital of Gannan Medical University, Ganzhou, Jiangxi Province, China

**Keywords:** Cystic-solid thyroid nodules, Meta-analysis, Ultrasound feature, Multimodal, Review, Malignant

## Abstract

**Background:**

The malignant risk of cystic-solid thyroid nodules may be underestimated in the ultrasound assessment.

**Objective:**

This systematic review and meta-analysis aimed to evaluate the value of multimodal ultrasound characteristics in the malignant risk assessment of cystic-solid thyroid nodules.

**Methods:**

We conducted a comprehensive search of PubMed, Web of Science, and Cochrane Library databases for studies depicting the ultrasound characteristics of cystic-solid thyroid nodules published prior to October 2023. The Review Manager 5.4 software was utilized to evaluate the ultrasound features suggestive of malignancy and to determine their sensitivity and specificity. Additionally, the Sata 12.0 software was utilized to construct summary receiver operating characteristic curves (SROC), estimate the area under the curve (AUC), and evaluate any potential publication bias.

**Results:**

This review included 16 studies comprising 5,655 cystic-solid thyroid nodules. Nine ultrasound features were identified as risk factors for tumor malignancy. Among the ultrasound features, microcalcification in the solid portion, heterogeneous hypoenhancement on Contrast-Enhanced Ultrasound (CEUS), and sharp angles in the solid portion exhibited higher malignant predictive value in cystic-solid thyroid nodules, with AUC values of 0.91, 0.84, and 0.81, respectively.

**Conclusion:**

Our findings indicate that microcalcification and sharp angles in the solid part of the nodule, along with heterogeneous hypoenhancement on contrast-enhanced ultrasound (CEUS), can better predict malignant cystic-solid thyroid nodules.

The systematic review and meta-analysis was registered prospectively in the International Prospective Register of Systematic Reviews (No. CRD42024602893).

## 1 INTRODUCTION

The thyroid, one of the largest endocrine glands in the human body, weighs approximately 20-30 grams in adults. Thyroid disorders are relatively common, affecting about 4%-7% of the population. Most thyroid abnormalities are asymptomatic and maintain normal hormone secretion [1]. Of note, approximately 5% of the population presenting with nodules are detectable through palpation [2]. Moreover, these nodules are often incidentally detected through ultrasonography, with a detection rate of up to 70%, and the incidence rate tends to increase with age. Cystic-solid thyroid nodules (CSTNs), which comprise both solid and cystic components, have a prevalence ranging from 15.0% to 53.8% [3]. In recent years, the prevalence of thyroid cancer within pathologically confirmed CSTNs has increased, accounting for 20% to 25% [4]. Various Thyroid Imaging Reporting and Data System (TI-RADS) [5-9] classifications focus primarily on solid thyroid nodules and have highlighted that several ultrasound features are significantly associated with malignancy. These include vertical position, indistinct margins, microcalcifications, and hypoechoic appearance. However, there has been a paucity of research assessing the diagnostic significance of ultrasound features in evaluating CSTNs. Despite conducting preliminary analyses, several studies have overlooked the use of contrast-enhanced ultrasound (CEUS), potentially affecting the outcomes and conclusions [10]. Additionally, clinicians often perceive CSTNs as having a lower risk of malignancy. However, we believe more attention should be given to assessing the risk of malignancy in CSTNs. To that end, we conducted a meta-analysis to evaluate the predictive value of multimodal ultrasound features in assessing CSTNs.

## 2 MATERIALS AND METHODS

### 2.1 Literature Search

This meta-analysis adhered to the Preferred Reporting Items for Systematic Reviews and Meta-Analyses (PRISMA) guidelines [11]. We gathered relevant literature from PubMed, Web of Science, and the Cochrane Library databases, using search terms such as “cystic-solid thyroid nodules” and “ultrasonography” or related terms like “sonography,” “ultrasonic,” or “ultrasound.” The search encompassed articles from the inception of the databases to October 31, 2023. We identified a total of 612 articles, with 327 from Web of Science and 285 from PubMed. No relevant studies were found in the Cochrane Library. We managed the literature using EndNote X8, removing duplicate articles manually.

The inclusion criteria were as follows: (1) Studies that focused on the ultrasound features of cystic-solid thyroid nodules; (2) Histopathologic or fine-needle aspiration (FNA) cytologic findings were used as the gold standard for diagnosis; (3) Studies assessing the diagnostic value of ultrasound features for evaluating cystic-solid thyroid nodules; and (4) Retrospective or prospective study designs.

Meanwhile, the exclusion criteria were as follows: (1) Studies with unclear diagnostic classifications or the lack of specific sonographic features for cystic-solid thyroid nodules; (2) Insufficient or problematic data needed for completing the 2x2 diagnostic tables; and (3) There were cases of inappropriate deletion of studied cases from the included literature.

Eventually, 16 studies were included, as depicted in the study selection flowchart in Fig. (**1**) [4, 12-26].

### 2.2 Data Extraction

The data extracted from the literature comprised: (1) Study characteristics, including first author, year of publication, study design, and inclusion period; and (2) Basic patient characteristics, including sex, age, and the number and quantity of malignant nodules. Two experts independently conducted the literature review and data extraction process to ensure consensus.

### 2.3 Literature Evaluation Criteria

The quality of the included studies was assessed using the Quality Assessment Tool for Diagnostic Accuracy Studies-2 (QUADAS-2). Each study was evaluated for quality and categorized as “yes,” “no,” or “unclear.” A rating of “yes” indicated that the criteria were met, “no” indicated that the criteria were not met or not mentioned, and “unclear” indicated that information from the literature was insufficient [27]. Additionally, the studies were also assessed for risk of bias and applicability to the research question. Ratings of “low,” “high,” or “unclear” were assigned to indicate the level of applicability and risk of bias. Any disagreements were resolved through discussion.

### 2.4 Statistical Analysis

Statistical analysis was performed using RevMan 5.4 and Stata 12.0 software. We employed a risk-of-bias assessment diagram to evaluate potential biases in the included literature. The sensitivity and specificity of various ultrasound features were analyzed, and these measures were quantitatively merged to generate a summary receiver operating characteristic curve (SROC). We calculated sensitivity and specificity along with their 95% confidence intervals (CI) for identifying the risk of malignant nodules. To assess heterogeneity among studies, we employed the Q test and the heterogeneity test, calculating the I^2^ inconsistency index. An I^2^ value ≥ 50% indicated significant heterogeneity, leading to the application of a random-effects model for meta-analysis. Conversely, in the absence of heterogeneity, a fixed-effects model was used. To explore potential publication bias and conduct sensitivity analysis, Deeks' funnel plots for different diagnostic indicators were employed. Forest plots were generated to illustrate the 95% CI and *p* value of the results, facilitating the evaluation of meta-analysis outcomes. Statistical significance was considered at *p* < 0.05.

## 3 RESULTS

### 3.1 Literature Search Results and Basic Information

Initially, we identified 612 studies through a search of three databases. Following the removal of 291 duplicates, we screened the titles and abstracts of the remaining 321 articles. Among these, 275 were excluded for various reasons: 244 were irrelevant or did not meet the eligibility criteria, and 31 were case reports. After a thorough review of the 46 remaining eligible articles, an additional 30 were excluded based on specific criteria: 20 articles either did not directly pertain to our study's topic or lacked complete information, 9 were review or commentary pieces, and 1 did not provide sufficient data for constructing 2×2 tables. Consequently, 16 articles met all the requirements and were included in our meta-analysis.

The included studies consisted of a total of 5,655 cases of cystic-solid thyroid nodules. Of these,779 were classified as malignant, while 4,876 were considered benign thyroid nodules. The sample sizes of the included studies ranged from 60 to 2,560 participants. Notably, these 16 articles provided detailed information on the effectiveness of various ultrasound characteristics in evaluating the risk of malignancy in cystic-solid thyroid nodules. The gold standard for diagnosis was either histopathologic or FNA cytologic findings of the thyroid. The basic characteristics of the included literature are summarized in Table **1**.

### 3.2 Quality Assessment

**Fig. (2):**
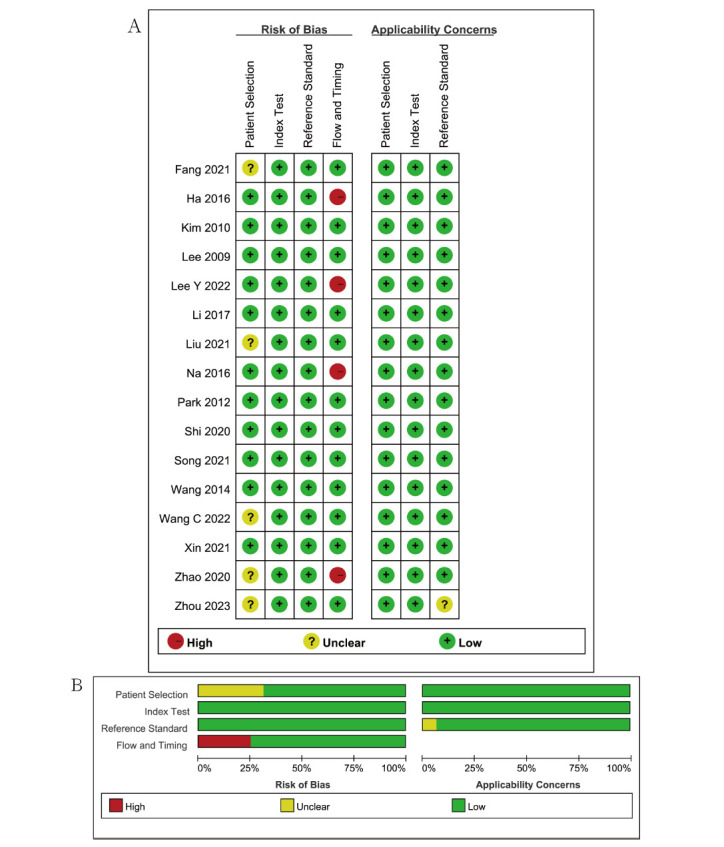
Outcome of QUADAS-2 for the included studies. (**A**) Risk-of-bias summary. (**B**) Risk-of-bias graph. Symbols: (+), low risk of bias; (?), unclear risk of bias;(-), high risk of bias.

According to the results of the QUADAS-2 questionnaire, all included studies exhibited a low risk of bias and were deemed to be of high quality. This was further confirmed using the literature risk of bias evaluation graph and the literature risk of bias summary graph (Fig. **2**).

### 3.3 Evaluation of the Diagnostic Performance of Ultrasound Features

**Fig. (3):**
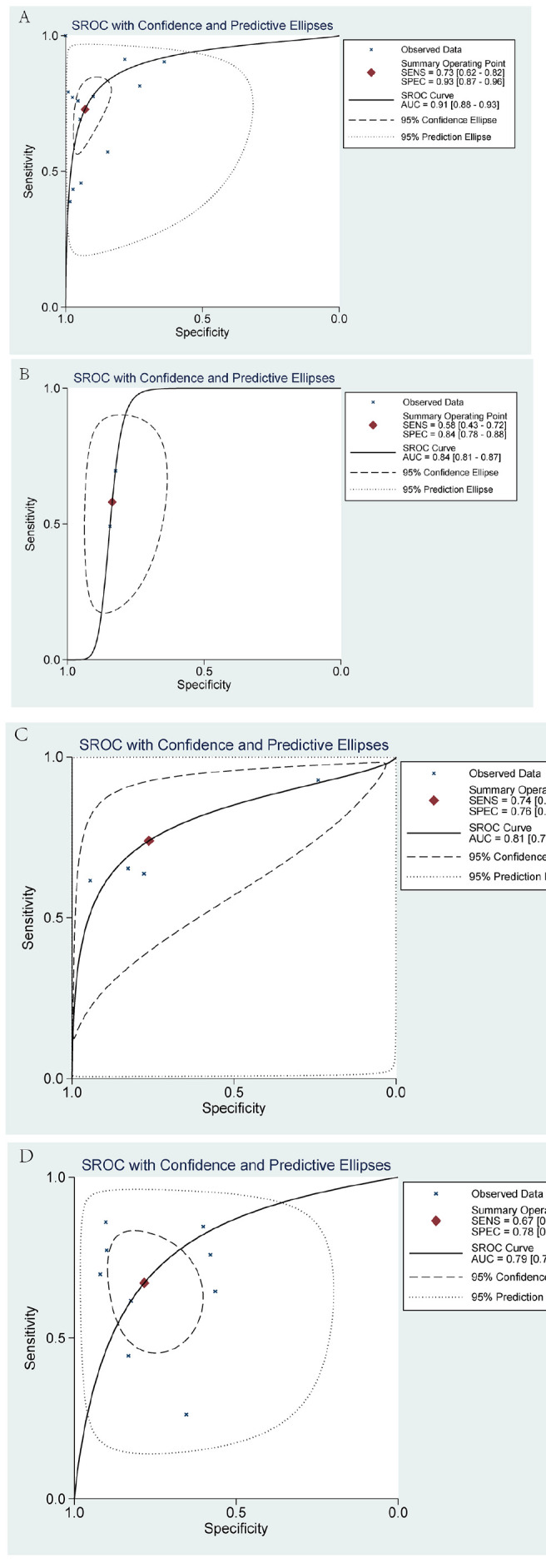
Summary receiver operator characteristic curve (SROC) with area under the curve (AUC) of four sonographic features in diagnosing cystic-solid thyroid nodules. **A**: microcalcification, **B**: heterogeneous low enhancement on contrast-enhanced ultrasound, **C**: sharp angle, **D**: eccentric position.

**Fig. (4):**
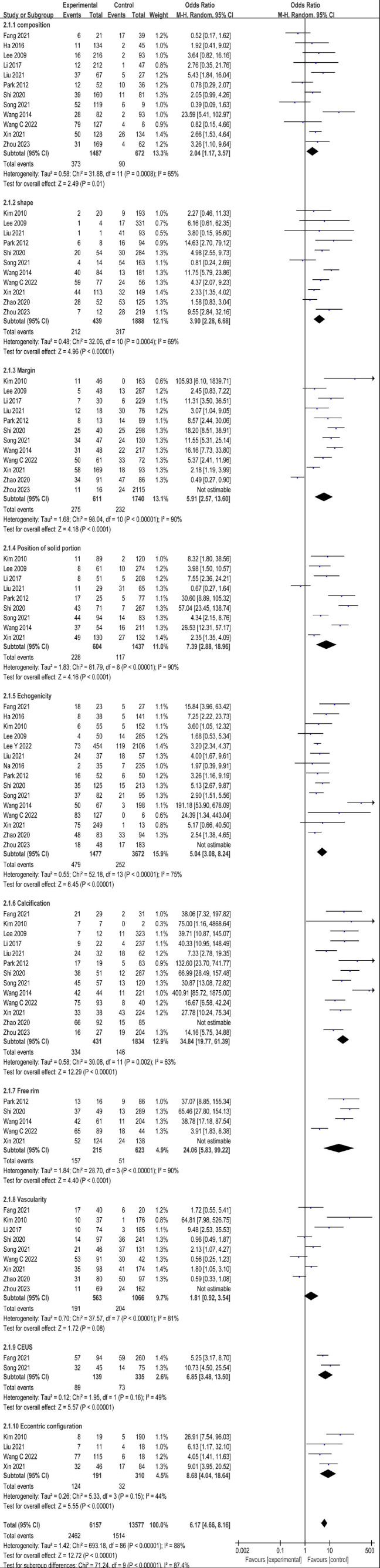
Odds ratio and respective 95% confidence intervals of ultrasound features of cystic-solid thyroid nodules.

**Fig. (5):**
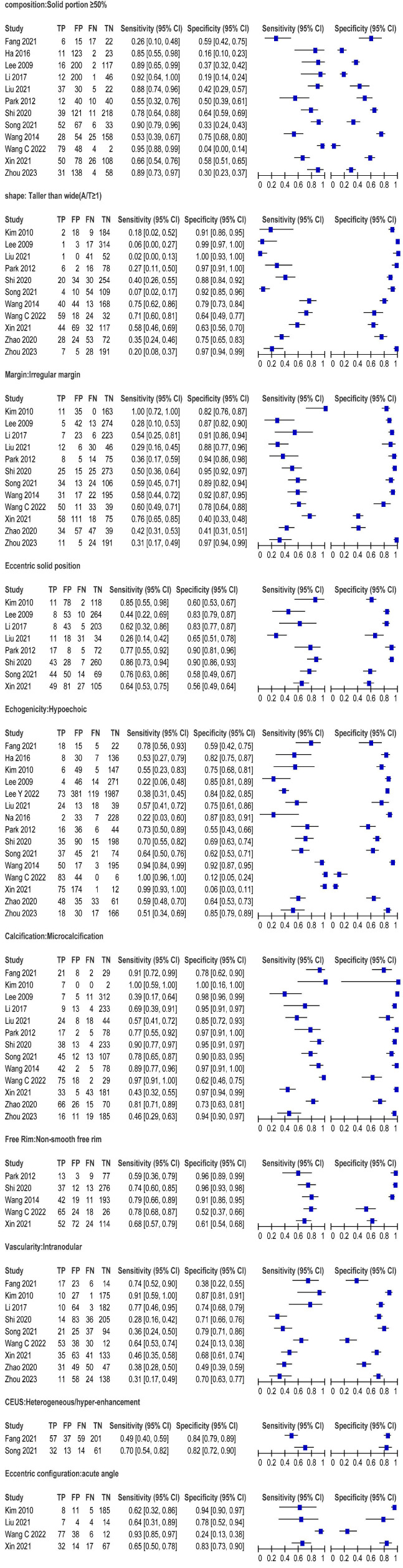
Forest plots of pooled sensitivity and specificity of ultrasound. **Note**: FN, false-negative; FP, false-positive; TN, true-negative; TP, true-positive.

Through our analysis, several ultrasound features indicate suspicion of malignancy in cystic-solid thyroid nodules, particularly those exhibiting a taller-than-wider shape and irregular margins (*p* < 0.05). Other malignant characteristics include an eccentric solid structure, non-smooth free edges, low echogenicity, sharp angles, microcalcifications, and heterogeneous hypoenhancement on CEUS. The presence of microcalcifications, sharp angles, and heterogeneous hypoenhancement indicates a higher likelihood of malignancy, with respective AUC values of 0.91 (95% CI: 0.88-0.93), 0.81 (95% CI: 0.77-0.84), and 0.84 (95% CI: 0.81-0.87) (Fig. **3**). The overall odds ratio (OR) values for all included suspicious malignant ultrasound features ranged from 1.81 to 34.84. All other malignant ultrasound features, except for the blood supply of the solid component of the thyroid nodule (*P* = 0.08), exhibited statistically significant differences (*p* <0.05) (Fig. **4**). Among the 16 included studies, the combined sensitivity and specificity for predicting suspected malignancy of cystic-solid thyroid nodules were generally good for all malignant ultrasound features, except for nodules with a solid component ≥50% (Fig. **5**).

The included study exhibited significant heterogeneity in terms of ultrasound features, leading to the subdivision of these features into 10 subgroups for further subgroup analysis. Cystic-solid thyroid nodules with a sharply angled solid portion were the only exception, as their ultrasonographic features changed from heterogeneity to homogeneity (I^2^ = 44%). However, for the vast majority of suspected malignancies, the heterogeneity of their ultrasonographic features remained stable and did not change significantly. Therefore, we employed the random-effects model to analyze these features and generated the corresponding SROC. The SROC for each suspected malignancy predictive ultrasound feature is depicted in Table **2**. The findings demonstrated high diagnostic accuracy and a low occurrence of false negatives and false positives, particularly regarding solid part microcalcification, acute angle, and inhomogeneous hypoenhancement.

**Fig. (6):**
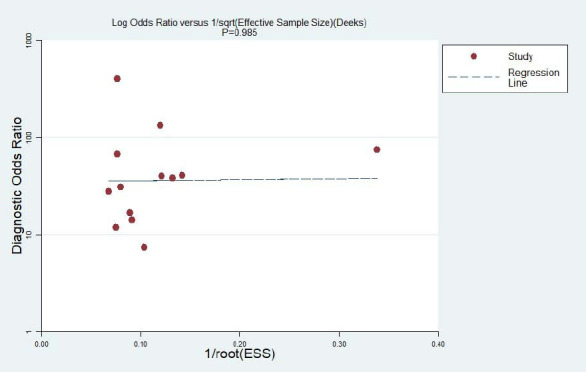
Deeks' funnel plot for publication of bias assessment.

We performed a sensitivity analysis by eliminating each study separately in order to see whether other factors affected the findings of these investigations. Interestingly, there was a notable change in heterogeneity when the Wang *et al* [23]. trial was excluded, with the I^2^ falling from 74% to 12%.. Additionally, no substantial publication bias was observed for various ultrasound characteristics, as indicated by the Deeks funnel plot (*p* > 0.05) (Fig. **6**).

## 4 DISCUSSION

The identification of benign and malignant cystic-solid thyroid nodules, based on ultrasound features, is of critical importance clinically. Consequently, we conducted a comprehensive systematic review and meta-analysis to assess the predictive value of ultrasound characteristics in predicting the risk of malignancy of cystic-solid thyroid nodules. In the studies we reviewed, there was a notable variation in the reported risk of malignancy for these nodules, with percentages ranging from 3.3% to 62.4%. Further examination of earlier research revealed that the inclusion of many cystic thyroid nodules with a TI-RADS classification of ≥4a was responsible for the elevated malignancy risk of 62.4% reported by Wang *et al*. [13]. This resulted in an abnormally high prevalence of malignant cystic-solid thyroid nodules in their study. Upon exclusion of this study, the prevalence of malignant cystic-solid thyroid nodules ranged from 3.3% to 44.7%, which still indicates a significant variation and poses a challenge in accurately diagnosing these nodules.

For this meta-analysis, we selected 16 studies comprising 5,655 cystic-solid thyroid nodules to assess the significance of suspected malignant ultrasound features in predicting nodule malignancy risk. Our findings revealed that the solid component of the nodule exhibited microcalcification, heterogeneous hypoenhancement on CEUS, and a sharp angle between the solid and cystic portions, all of which were highly indicative of malignant potential in cystic-solid thyroid nodules. The AUC values for microcalcification, heterogeneous hypoenhancement on CEUS, and a sharp angle between the solid and cystic portions were 0.91 (95% CI: 0.88-0.93), 0.84 (95% CI: 0.81-0.87), and 0.81 (95% CI: 0.77-0.84) respectively. Additionally, the analysis of the 16 included literature papers revealed that the combined specificity of the qualitative analysis of suspected malignant ultrasound features was generally good, except for the solid portion ≥50% of these features. The highest combined specificity was observed for microcalcifications, with a specificity of 0.93 (95% CI: 0.87-0.96), followed by taller-than-wide shapes and heterogeneous hypoenhancement, with specificities of 0.91 (95% CI: 0.82-0.97) and 0.89 (95% CI: 0.66-0.97), respectively. Our study differs from that of Shi *et al*. [20] because we analyzed the solid part of the thyroid nodule with an eccentric structure by dividing it into two ultrasound characteristics: positional eccentricity and the acute angle formed by the solid part. These findings introduce an additional ultrasound feature that can be used to predict the malignancy risk of CSTNs. Moreover, we included CEUS features in the malignant risk assessment of cystic-solid thyroid nodules. The findings suggest that CEUS features heterogeneous hypoenhancement holds significant value in assessing the malignant risk, with an AUC of 0.84 (95% CI: 0.81-0.87). Furthermore, this highlights the reliability of CEUS in determining the malignant risk of cystic-solid thyroid nodules, consistent with previous studies [28, 29]. Nevertheless, due to the limited literature on the use of CEUS for CSTNs, future research is warranted for a more comprehensive analysis.

In the present study, we performed a sensitivity analysis of the included literature using a case-by-case exclusion approach. After excluding the study by Wang *et al*. [23], we observed a significant decrease in the heterogeneity of the remaining articles. The I^2^ statistic decreased to 12% from the initial value of 74%, indicating that the study could have significantly contributed to the observed heterogeneity. Of note, the study by Wang *et al*. included a high percentage of cases (38.1%, 101/265) exhibiting ultrasonographic features of honeycomb spongy structures. However, these structures typically indicate benign CSTNs, with a probability of malignancy of less than 1% [30]. Therefore, the above discrepancy might be the potential source of heterogeneity.

We plotted the SROC for ultrasound features suspected to be malignant. Herein, the AUCs of eight ultrasonographic features (microcalcification, heterogeneous hypoenhancement, sharp angle between the solid and cystic portions, eccentric configuration, hypoechoic, irregular margins, taller-than-wide shape, and non-smooth free edges) were all greater than 0.7, indicating that these ultrasonographic features might have high predictive value. Notably, the AUC for microcalcification was greater than 0.9, indicating a high malignancy predictive value. This is inconsistent with the study conducted by Shi *et al*. [20]. They identified “taller-than-wide” as the strongest independent predictor of malignant thyroid nodules. The discrepancy might stem from differences in the number of studies included; they had fewer studies and did not incorporate any relevant studies that utilized CEUS.

Interestingly, our study found that Bethesda III-IV thyroid nodules carry a malignancy risk of 15%-40%, overlapping with the 20%-25% risk associated with cystic-solid thyroid nodules. Research shows incidental malignancy in 18.42% of Bethesda III multinodular goiter cases and 20.13% in solitary nodular goiter cases [31], compared to 1.53% in Bethesda II cases [32]. The most common malignancy is papillary thyroid carcinoma, which we surmise may stem from the high heterogeneity of nodule composition and inconsistencies in Bethesda reporting, leading to an underestimation of malignancy risk. The surgical management of differentiated thyroid cancer remains a topic of debate, particularly concerning the choice between total thyroidectomy and subtotal thyroidectomy. A key point of contention is whether total thyroidectomy increases the risk of early postoperative complications compared to subtotal thyroidectomy. Recent findings by Greek scholars suggest no significant difference in complications such as hypocalcemia, hematoma, infection, or temporary recurrent laryngeal nerve palsy between the two procedures [33]. These findings indicate that total thyroidectomy can be performed safely without increasing early complication risks, providing valuable insights into this ongoing debate.

Several significant limitations must be acknowledged. Firstly, the studies included in this research are primarily from China and South Korea, which may restrict the generalizability of our findings to other regions. Secondly, not all 16 studies included in the analysis contained all 10 suspicious malignant ultrasound features, and different diagnosis standards were employed in various literature. Thirdly, the number of studies included in the analysis of multimodal ultrasound with CEUS was limited, with only two studies. Fourthly, this study included publications in English only, potentially introducing bias by excluding articles in other languages.

## CONCLUSION

The present meta-analysis comprehensively examined relevant literature regarding the use of ultrasound features to assess the malignant risk of CSTNs. Importantly, our findings revealed that microcalcifications, sharp angles between the solid and cystic portions, and heterogeneous hypoenhancement on CEUS could more accurately predict malignancy risk in cystic-solid thyroid nodules.

## Figures and Tables

**Fig. (1):**
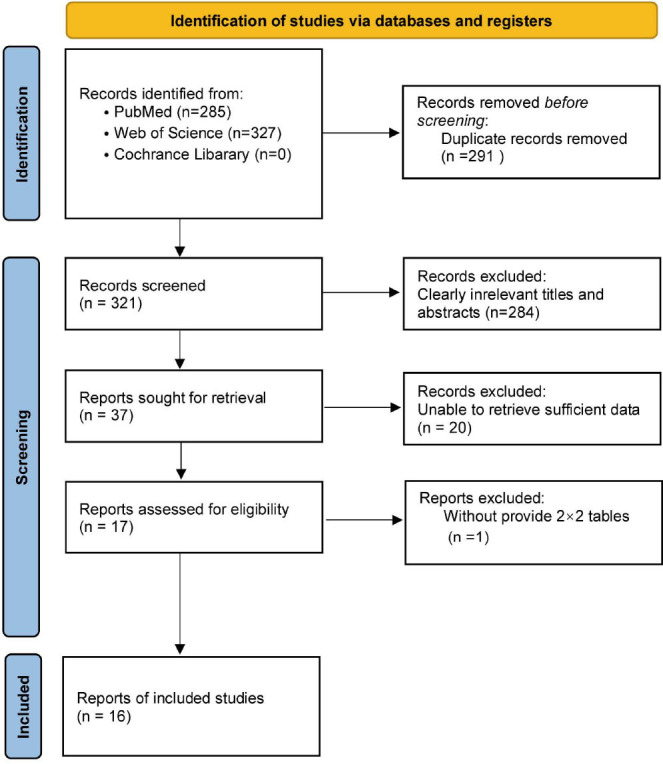
Flowchart of the study selection process.

**Table 1:** Basic information of the included studies.

First Author/Refs	Year	Country	Design	Sex (M/F)	Nodules (n)	Malignant Nodules (n)	Period of Enrollment	Included Nodules (n)
Li W [4]	2017	China	Prospective	44/237	281	13	2004-2012	259
Zhou T [12]	2023	China	Retrospective	73/212	301	35	2020-2021	231
Wang CY [13]	2022	China	Retrospective	59/74	133	83	2016-2020	133
Lee YJ [14]	2022	Korea	Retrospective	888/4101	2560	192	2015	2560
Xin Y [15]	2021	China	Retrospective	82/263	345	76	2017-2020	262
Song Q [16]	2021	China	Retrospective	37/140	177	58	2016-2019	177
Liu Y [17]	2021	China	Retrospective	23/68	94	42	2016-2017	94
Fang F [18]	2021	China	Retrospective	8/52	60	23	2017-2021	60
Zhao HN [19]	2020	China	Retrospective	44/130	200	81	not available	177
Shi YZ [20]	2020	China	Retrospective	91/216	338	50	2012-2017	338
Na DG [21]	2016	Korea	Prospective	207/635	270	9	2010-2011	270
Ha EJ [22]	2016	Korea	Prospective	156/594	187	13	2013-2015	179
Wang X [23]	2014	China	Retrospective	126/139	265	53	2006-2013	265
Park JM [24]	2012	Korea	Retrospective	23/79	102	22	2003-2010	102
Kim DW [25]	2010	Korea	Prospective	41/155	213	11	2008-2009	213
Lee MJ [26]	2009	Korea	Retrospective	34/349	392	18	2002-2003	335

**Table 2:** Diagnostic performance of each sonographic feature.

Sonographic Features	SROC AUC	95% CI	Sensitivity	95% CI	Specificity	95% CI	Deek *P*-value
Solid portion ≥50%	0.65	0.61-0.69	0.81	0.68-0.89	0.39	0.26-0.53	0.171
Taller-than-wide shape	0.72	0.68-0.75	0.26	0.12-0.46	0.92	0.82-0.97	0.419
Irregular margin	0.72	0.68-0.76	0.51	0.40-0.62	0.86	0.76-0.93	0.299
Eccentric solid position	0.79	0.75-0.83	0.67	0.53-0.78	0.78	0.68-0.83	0.865
Hypoechoic	0.75	0.71-0.79	0.72	0.51-0.86	0.68	0.51-0.81	0.754
Microcalcification	0.91	0.88-0.93	0.73	062-0.82	0.93	0.87-0.96	0.985
Non-smooth free rim	0.75	0.71-0.78	0.73	0.67-0.78	0.86	0.66-0.95	0.702
Acute angle	0.81	0.77-0.84	0.74	0.52-0.88	0.76	0.43-0.93	0.581
Heterogeneous hypoenhancement	0.84	0.81-0.87	0.58	0.43-0.72	0.84	0.78-0.88	0.936
Intranodular Vascularity	0.61	0.56-0.65	0.52	0.38-0.66	0.64	0.51-0.65	0.051

**Abbreviations: **SROC: summary receiver operator characteristic curve; AUC, area under the curve; CI: confidence interval.

## Data Availability

The original contributions presented in the study are included in the article/supplementary material. Further inquiries can be directed to the corresponding author [J.Y].

## LIST OF ABBREVIATIONS

QUADAS: Quality Assessment of Diagnostic Accuracy Studies
SROC: Summary Receiver Operating Characteristic Curve
AUC: Area Under the SROC
CEUS: Contrast-enhanced Ultrasound
CSTNs: Cystic-solid Thyroid Nodules
TI-RADS: Thyroid Imaging Reporting and Data System
PRISMA: Preferred Reporting Items for Systematic Reviews and Meta-Analyses
CI: Confidence Intervals
FNA: Fine-needle Aspiration
OR: Odds Ratio

## STANDARDS OF REPORTING

PRISMA Guidelines and methodology were followed.

## SUPPLEMENTARY MATERIALS

PRISMA checklist is available as supplementary material on the publisher’s website along with the published article.
